# Efficacy of Application of Pseudocolor Filters in the Detection of Interproximal Caries

**DOI:** 10.5681/joddd.2010.020

**Published:** 2010-09-16

**Authors:** Maryam Zangooei Booshehry, Abdolrahim Davari, Fatemeh Ezoddini Ardakani, Mohammad Reza Rashidi Nejad

**Affiliations:** ^1^ Assistant Professor, Department of Oral and Maxillofacial Radiology, Faculty of dentistry, Shahid Sadoughi University of Medical Sciences, Yazd, Iran; ^2^ Associate Professor, Department of Operative Dentistry, Faculty of Dentistry, Shahid Sadoughi University of Medical Sciences, Yazd, Iran; ^3^ Associate Professor, Department of Oral and Maxillofacial Radiology, Faculty of Dentistry, Shahid Sadoughi University of Medical Sciences, Yazd, Iran; ^4^ Dentist, Faculty of Dentistry, Shahid Sadoughi University of Medical Sciences, Yazd, Iran

**Keywords:** Dental caries, digital radiography, pseudocolor

## Abstract

**Background and aims:**

The aim of the present study was to compare the effect of application of an image processing mode of a colorizer on the efficacy of the detection of interproximal carious lesions viewed in direct digital radiography.

**Materials and methods:**

A total of 102 proximal surfaces of extracted human premolars on direct digital images were evaluated by three observers with and without the application of pseudocolor filter. The teeth were sectioned and viewed microscopically to determine the gold standard. The kappa value agreement ratios were calculated.

**Results:**

Sensitivity and specificity values for normal digital and colorized images were 66.7%, 60%, 80.5%, and 50%, respectively. However, there were no statistically significant differences between the two types of images (P = 0.12).

**Conclusion:**

In this study application of pseudocolor filter on digital radiographic images failed to result in significantly improved caries detection.

## Introduction

Dental caries, a chronic infectious disease, is very common and affects 95% of the population; it is still a major cause of tooth loss.^1^ The growing sophistication in available interventions for prevention and non-surgical treatment of dental caries is matched by a similar increase in the available methods for diagnosis of carious lesions.^2^ Diagnosis of posterior approximal carious lesions by means of bite-wing radiographs is an approved clinical method.^3^

Although radiographic examination by means of conventional dental films is still a useful diagnostic tool, radiography has many limitations, including the need for ionizing radiation, physical limitations based on anatomic considerations, and the high degree of inter- and intra-examiner variability.^4^

Direct digital sensors for intraoral radiography are very sensitive and their use may lead to significant reduction of exposure time. Most in vitro studies have shown similar results with direct digital radiography and conventional radiographic films for the detection of approximal caries.^3^

Shrout et al^5^ in 1996 showed that digital manipulation of captured images enhances caries detection accuracy; however, one of the three observers who could achieve this capability successfully was a maxillofacial radiologist.

In 1999 a study by Eickholz^6^ on application of different digital filters on bite-wing radiographs of extracted teeth reported the inability of FRICOM software filters to improve approximal caries diagnosis. Hack^7^ showed that contrast enhancement of digital images could improve approximal caries diagnosis. Gakenheimer^8^ reported the LOGICON software capability to improve caries diagnosis up to 20%.

Koob et al^3^ and Hack^9^ could not improve approximal caries diagnosis in their study by application of noise reduction and gray scale reversal, respectively.

However, information about the use of "pseduocolor" tool to improve direct digital radiographic detection of proximal caries has not been reported yet.^10^

## Materials and methods

We used teeth extracted during routine clinical treatment for our evaluation. The sample consisted of 51 unrestored teeth with non-cavitated interproximal surfaces based on visual inspection. The teeth had been stored in saline with thymol (1%) added to prevent bacterial growth. Tooth surfaces ranged from sound to discolored after cleaning, with white/brown discoloration.

Every three teeth were mounted in dental stone blocks, simulating the clinical situation where teeth would be in proximal contact. Each block had a code and a leaded marker, determining the mesial aspect of the teeth

###  Image acquisition

**Figure 1:**
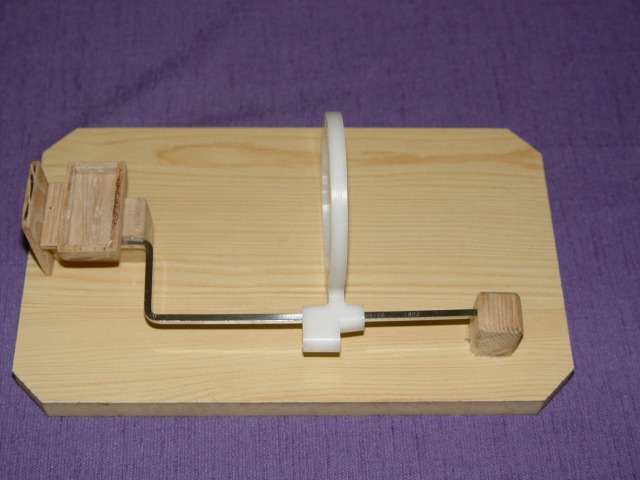
The optical bench which was constructed and used as a film holder.

In order to standardize projection geometry, an optical bench was constructed, consisting of a positioning ring (Rinn Corporation, USA) for the x-ray tube in combination with the corresponding film holder mounted on a wooden platform; a wooden box was placed near it for the placement of dental casts (Figure 1). By placing each dental cast in the corresponding box, projection geometry was obtained, in which the central ray passed orthogonally through the interproximal contact. The resultant focus-to-object distance was 15 cm. Digital images of the teeth were acquired by using a dental x-ray unit (ELITYS Trophy, TRX 708, CROISSY BEAUBOURG, France) for all exposures operating at 70 kVp, 8 mA, and 0.0.1 sec with 2.0 mm aluminum equivalent filtration.

Direct digital images were obtained with a direct digital sensor (RVG UI6, Trophy, Valle, France).

### Viewing sessions

Three observers (a maxillofacial radiologist, an operative dentistry specialist and a dentist) were recruited for this study. They were asked to score the presence or absence of caries in the proximal surfaces of the teeth.

The observers were instructed to assess only proximal surfaces coronal to the cemento-enamel junction. They also were told that any decalcification should be considered, regardless of its size, or its management strategy. Observers viewed images in 2 viewing sessions for the two imaging modes, with a 2-week interval.

###  Histological examination

Subsequent to imaging, the teeth were sectioned mesiodistally into two sections, using a ground section device (DEMCO Non-stop E6-230, USA). Tooth sections were examined under a stereomicroscope at ×20 magnification by two observers. The results were registered in a 2-point scale, in which 0 equals absence of caries and 1 equals presence of caries.

### Statistical analysis

Observers’ assessments using each of the imaging modes were compared with the baseline data to determine the diagnostic performance, using “kappa” correlation coefficient. Statistical significance was defined at α=0.05.

## Results

According to the microscopic assessment, 30 (29.4%) of a total of 102 evaluated interproximal surfaces were intact, whereas 72 (70.6%) exhibited carious lesions.

Inter-observer agreement according to correlation coefficient was computed too (Tables 1 and 2), which showed that inter-observer agreement between observers 2 and 3 was better than the inter-observer agreement of observers 1 and 2. Kappa correlation coefficient of observers demonstrated good observer agreement.

**Table 1:** Inter-observer agreement percentage and correlation coefficient of caries detection in gray scale mode of digital imaging

Observers	Agreement percent	Kappa correlation coefficient
1&2	67.6	0.33
1&3	85.2	0.70
2&3	74.5	0.40

**Table 2:** Inter-observer agreement percentage and correlation coefficient of caries detection in colored mode of digital imaging

Observers	Agreement percent	Kappa correlation coefficient
1&2	68.6%	0.27
1&3	60.7%	0.11
2&3	62.7%	0.38

There was a weak correlation between each of the imaging mode and gold standard, as shown in Table 3.

**Table 3:** Sensitivity, specificity, positive (PPV) and negative predictive values (NPV), agreement percentage and correlation coefficient with histology of each of the imaging modes

Imaging mode	Agreement percent with histology	NPV	PPV	Specificity	Sensitivity	Kappa correlation coefficient with histology
Gray scale	64.7%	42.8%	80%	60%	66.7%	0.24
Colored	71.5%	51.7%	72.2%	50%	80.5%	0.30

Sensitivities, specificities, and positive and negative predictive values were computed for each of the imaging modes and for each of the observers (Table 4).

**Table 4:** Sensitivity, specificity, positive (PPV) and negative predictive values (NPV), agreement percent and correlation coefficient with histology of each of the imaging modes for each of the observers

Parameters / Observers	Agreement percentage with histology	NPV	PPV	Specificity	Sensitivity	Kappa correlation coefficient with histology
Observer 1	62.7%	40.9%	79.3%	60%	63.9%	0.20
Observer 2	67.6%	46.3%	81.9%	63.3%	69.5%	0.54
observer 3	61.7%	40%	78.9%	60%	62.5%	0.19

Comparison of the correct answers as shown in Table 5 were in favor of no statistically significant differences between the two imaging modes (P = 0.12)

**Table 5:** Distribution of correct answers according to imaging mode

Colored /Gray scale	Negative	Positive	Total
Positive	23	48	71
Negative	15	16	31
Total	38	64	102

## Discussion

Radiography is still the diagnostic standard in the detection of inaccessible approximal caries, and presently conventional dental films are frequently replaced by digital imaging systems.^11^

In addition to many advantages of digital imaging, post-processing of the image is a point of interest, which means alteration of captured images with different software filters in order to improve the quality of the image or to analyze its contents.^12^ The rationale for the study was to provide evidence for the clinicians that post-processing filter of psudocolor has a diagnostic performance comparable to their well-known traditional gray scale views, which has not been approved yet.^11 , 12^

Extensive carious lesions are rarely misdiagnosed on a radiograph; therefore, in this study we used extracted teeth whose approximal surfaces were either intact or had discoloration without cavitations on visual inspection.^13^

The number of the teeth in our study was 52 and the number of the observers was 3, selected based on the report of Hintz et al.^14^

However, caries diagnosis is a contrast-dependent task and we were unable to find significant differences between gray-scale and colored images in this aspect, which might be explained by unfamiliarity of the clinician’s eyes with colored images, their conception, analysis and interpretation.^15 , 16 , 17^ Therefore, as shown in the results, inter-observer agreement in gray-scale images was better than this value in colored images.

## Conclusion

In conclusion, as shown previously by Koob,^3^ Shorut^5^ and Haak^7^ post-processing by digital images failed to result in significant improvements in the accuracy of caries detection. It is possible that alterations in pseudocolor filter in order to differentiate more densities from each other and operators' familiarity with colored images will make this tool efficient and easy to use.
